# The Glyoxalase System and Methylglyoxal-Derived Carbonyl Stress in Sepsis: Glycotoxic Aspects of Sepsis Pathophysiology

**DOI:** 10.3390/ijms18030657

**Published:** 2017-03-17

**Authors:** Thomas Schmoch, Florian Uhle, Benedikt H. Siegler, Thomas Fleming, Jakob Morgenstern, Peter P. Nawroth, Markus A. Weigand, Thorsten Brenner

**Affiliations:** 1 Department of Anesthesiology, Heidelberg University Hospital, 69120 Heidelberg, Germany; Thomas.Schmoch@med.uni-heidelberg.de (T.S.); Florian.Uhle@med.uni-heidelberg.de (F.U.); Benedikt.Siegler@med.uni-heidelberg.de (B.H.S.); Markus.Weigand@med.uni-heidelberg.de (M.A.W.); 2 Department of Medicine I and Clinical Chemistry, Heidelberg University Hospital, 69120 Heidelberg, Germany; Thomas.Fleming@med.uni-heidelberg.de (T.F.); Jakob.Morgenstern@med.uni-heidelberg.de (J.M.); Peter.Nawroth@med.uni-heidelberg.de (P.P.N.)

**Keywords:** sepsis, septic shock, metabolic stress, immunometabolism, Warburg effect, reactive carbonyl species, methylglyoxal, glyoxalase

## Abstract

Sepsis remains one of the leading causes of death in intensive care units. Although sepsis is caused by a viral, fungal or bacterial infection, it is the dysregulated generalized host response that ultimately leads to severe dysfunction of multiple organs and death. The concomitant profound metabolic changes are characterized by hyperglycemia, insulin resistance, and profound transformations of the intracellular energy supply in both peripheral and immune cells. A further hallmark of the early phases of sepsis is a massive formation of reactive oxygen (ROS; e.g., superoxide) as well as nitrogen (RNS; e.g., nitric oxide) species. Reactive carbonyl species (RCS) form a third crucial group of highly reactive metabolites, which until today have been not the focus of interest in sepsis. However, we previously showed in a prospective observational clinical trial that patients suffering from septic shock are characterized by significant methylglyoxal (MG)-derived carbonyl stress, with the glyoxalase system being downregulated in peripheral blood mononuclear cells. In this review, we give a detailed insight into the current state of research regarding the metabolic changes that entail an increased MG-production in septicemia. Thus, we point out the special role of the glyoxalase system in the context of sepsis.

## 1. Introduction

### 1.1. Sepsis: Definitions, Incidence, Outcome, and Economic Relevance

Sepsis, defined as a “life-threatening organ dysfunction caused by a dysregulated host response to infection” [1], is characterized by an increasing incidence within the last decades and remains one of the leading causes of death in intensive care units (ICUs) [2,3,4,5,6]. Although mortality rates declined [7] after the implementation of treatment protocols according to the recommendations of the Surviving Sepsis Campaign [8,9,10], overall mortality remains high with rates of 20%–50% causing considerable medical and economic challenges for both health systems and society [2,3,4,5,6].

### 1.2. Traditional Pathophysiology of Sepsis: PAMPs, PRRs and Reactive Metabolites

Although sepsis is caused by fungal, viral or bacterial infections, the dysregulated generalized host response ultimately leads to severe dysfunction of multiple organs and death [1]. While the underlying pathophysiological mechanisms have not been fully elucidated yet, the innate immune system seems to dominate the early phase of the disease. Pattern recognition receptors (PRR) on immune and endothelial cells recognize unspecific and highly conserved antigens before launching a diversified stress response. These unspecific and highly conserved antigens are called pathogen-associated molecular patterns (PAMPs) and include bacterial cell wall components, such as lipopolysaccharide (LPS) or peptidoglycan (PGN). The resulting cell activation leads to degranulation, phagocytosis, upregulation of adhesion molecules, the release of pro-inflammatory cytokines, such as interleukin 1 (IL-1), IL-6 and tumor necrosis factor α (TNF-α), in addition to the activation of complement pathways causing a pro-coagulatory state [11,12,13,14,15]. Furthermore, studies observed a massive formation of reactive oxygen (ROS; e.g., superoxide) as well as nitrogen (RNS; e.g., nitric oxide) species [11,12,13,14,15]. In addition to ROS and RNS, reactive carbonyl species (RCS) represent another crucial group of highly reactive metabolites [16], which has not been a focus in sepsis research until recently. However, RCS might be a crucial component of the observed changes resulting from the response to an infectious stimulus. In normal physiology, the inflammatory response is strictly limited to the infectious focus with a well-balanced production and elimination of radicals and cytokines [17]. In contrast, during sepsis the body enters a vicious cycle, resulting in a substantial imbalance of these physiological conditions. Altogether these changes have been termed as the systemic inflammatory response syndrome (SIRS) [18]. Although this term was abolished in the latest definition of sepsis [1], it accurately describes the clinical correlations of the early phase of the host response to an infectious stimulus, which is often accompanied by a hyperdynamic cardiovascular circulatory state, fever and changes in the white blood cell count [14,19]. At the same time a counter-regulatory anti-inflammatory response is launched, including an increase in IL-10 release and a remarkable reprogramming of immune cells, partially resulting in immune cell apoptosis and autophagy [20]. Although this compensatory anti-inflammatory response syndrome (CARS) is initiated shortly after the onset of sepsis and its signaling cascades might have an influence on the metabolism in the early phase of sepsis, the first days of the disease are still characterized by the above mentioned pro-inflammatory disease state. The concomitant profound metabolic changes are orchestrated by an interaction of immunoinflammatory and neuroendocrine stress responses [21].

## 2. Different Aspects of the Stress Response in Sepsis

### 2.1. Neuroendocrine Reactions in Sepsis

The neuroendocrine response is dominated by activation of the sympathetic nervous system and the hypothalamic-pituitary axis [21]. Among the variety of “stress” hormones that are consequently released, the most important are probably cortisol, epinephrine, norepinephrine, vasopressin in addition to both insulin and glucagon [13,14,15] (Table 1).

Consequently, the first phase of sepsis is often characterized by a hyper-hemodynamic state. Moreover, the excessive release of these hormones result in the metabolic status being substantially changed. The induction of both hepatic gluconeogenesis, glycogenolysis and glycolysis is predominantly triggered by therapeutic and endogenous epinephrine and norepinephrine [22]. Furthermore, norepinephrine increases the supply of glycerol by activating peripheral lipolysis. Cortisol additionally worsens the hyperglycemic state by not only stimulating hepatic gluconeogenesis, but also inducing insulin resistance and thus decreasing glucose uptake by muscles and adipose tissue [22,23]. This induced insulin resistance is further amplified by inflammatory mediators, predominantly TNF-α, IL-1, IL-6 and C-reactive protein [22]. 

### 2.2. Metabolic Changes in Sepsis: Hyperglycemia, Insulin Resistance and Respiratory Chain Uncoupling

The immunoinflammatory and neuroendocrine responses to an infectious stimulus lead to a distinct catabolic metabolism co-existing with insulin resistance. As a result, hyperglycemia can frequently be observed in patients with sepsis [22,24]. It is important to note that hyperglycemia, especially severe hyperglycemia, is known to be associated with increased mortality and morbidity in critically ill patients [25,26,27,28,29]. Nevertheless, a large randomized trial targeting intensive glucose control as a means of normalizing blood sugar (81–101 vs. >180 mg/dL) observed an increased mortality in patients with sepsis [30]. Although this increased mortality was suspected to be due to the higher rate of accidental overdosing causing hypoglycemic episodes, it is possible that the excess supply of glucose is a physiological adaption to stress in critical illness. This might be due to the fact that mitochondrial glucose utilization is impaired in many tissues during systemic inflammation [31,32]. One reason might be that the mitochondrial electron transport chain is uncoupled or otherwise altered in these tissues during systemic inflammation [32], with a subsequent large dependence on glycolysis for their energy supply. However, since glycolysis is less effective in producing adenosine triphosphate (ATP) in comparison to oxidative phosphorylation (glycolysis produces only 2 ATP compared with 30 ATP from the citrate circle), large amounts of glucose are temporarily needed in order to cover energy demands. In parallel, a deranged microcirculation further impedes the glucose supply in sepsis. Therefore, the host is forced to provide high blood glucose levels in order to maintain a sufficient glucose gradient, which acts as the driving force for transmembranous glucose transporters (GLUT) [22,23,33]. Both non-insulin-dependent glucose uptake via GLUT1, 2 or 3 and insulin-dependent glucose uptake via GLUT4 rely on concentration gradients, necessitating higher glucose concentrations in the bloodstream than in the cells [34]. While peripheral tissues develop a significant insulin resistance during the course of sepsis due to the downregulation of GLUT4 [35] and the impairment of post-receptor signaling pathways (via the phosphorylation of the insulin receptor, insulin receptor substrate 1 (IRS-1) and MAP kinase) [36], GLUT1 seems to be upregulated in brain cells of mice following thermal injury or infection with *Pseudomonas aeruginosa*, [37]. Similarly, white blood cells increase the expression of GLUT1, 3 and 4 after being activated [38]. In conclusion, a glucose balance that is adaptive to the necessities of the first phase of the immune response to an infectious stimulus is achieved by high blood sugar levels during systemic inflammation, upregulation of GLUT1 and GLUT3 in addition to the downregulation of GLUT4. These changes facilitate the redirection of glucose away from peripheral tissues towards immune cells and the nervous system [23].

Since hexokinase (the rate limiting enzyme of glycolysis) is saturated under physiological glucose concentrations, glycolytic flux can only be increased via one of the following mechanisms: (1) increase of the hexokinase activity; (2) increase of hexokinase expression; and (3) hexokinase independent glucose-6-phospate generation. All three mechanisms might be of relevance in sepsis, although there might be tissue-specific differences. First, an increase in hexokinase activity was observed in the renal cortical cells of mice following LPS exposure [39]. A similar increase in hexokinase activity was observed in immune cells [40]. Secondly, LPS is known to activate the hypoxia-inducible factor 1α (HIF1α), which in turn induces the upregulation of several glycolytic enzymes, including hexokinase, in immune cells, heart and peripheral tissues [41,42]. Finally, the metabolic shift in sepsis towards a stress state includes high cortisol levels resulting in increased rates of hepatic gluconeogenesis [13], which might be an alternative source of glucose-6-phospate in sepsis. 

### 2.3. Immunometabolism in Sepsis: The “Warburg Effect” and Its Consequences for Immune Cells

**Figure 1:**
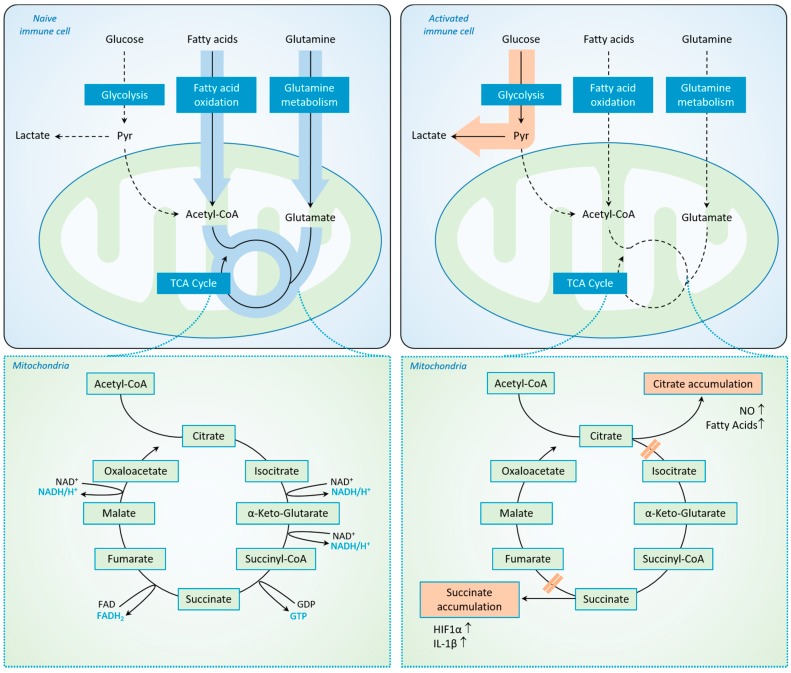
The “Warburg effect” in activated immune cells. Visualization of the interaction of glycolysis and the tricarboxylic acid cycle (TCA) pathway in naive (**left side**) and activated (**right side**) immune cells. While energy production relies on the TCA in native immune cells (solid arrows highlighted in blue) and Glycolysis is of minor importance (dashed arrows **left side**), the TCA is blocked on two breaking points in activated immune cells, which results in citrate and succinate accumulation. As a result, energy production in activated immune cells relies on Glycolysis (solid arrows highlighted in orange) while the TCA is of minor importance (dashed arrows). Upward arrows symbolize increased levels. Abbreviations: Pyr = Pyruvate, NADH = Nicotinamide adenine dinucleotide (NAD^+^ in its oxidated form), GDP/GTP = Guanosine diphosphate/Guanosine triphosphate, HIF1α = Hypoxia-inducible factor 1-α, IL-1β = Interleukin 1 β, NO = nitric oxide.

The metabolic switches in immune cells during systemic inflammation are of special importance. Aside from changes in utilization of fatty and amino acids, the changes in glucose metabolism seem to be of special importance for the immune response [43]. Thereby, the switch from mitochondrial oxidative phosphorylation to cytosolic glycolysis being the major source of ATP constitutes a hallmark change in all activated immune cells. This phenomenon, known as the “Warburg effect”, was first described in tumor cells [44] and the underlying concept was subsequently extended to include immune cells, such as monocytes, macrophages, dendritic cells [45,46], B cells [47], T cells [48] and natural killer cells [49] during activation [50] (Figure 1).

Although glycolysis is quite inefficient with regard to ATP production in comparison to the oxidative phosphorylation, it provides valuable biosynthetic intermediates and co-enzymes urgently needed for core activities of activated immune cells like cell growth, proliferation or cytokine production [43]. Important examples of processes supported by glycolysis include the reduction of nicotinamide adenine dinucleotide (NAD^+^) to NADH, which is used by numerous enzymes as a co-factor, and the provision of biosynthetic intermediates for the synthesis of ribose needed for nucleotide production [43]. Through the latter pathway, enhanced glycolysis is intimately linked to an upregulation of the pentose phosphate pathway, providing not only nucleotide intermediates but also NADPH needed for the so-called respiratory burst (discussed below). In a recent review [43], O’Neill et al. gathered molecular insights into the signaling pathways, which are involved in the upregulation of glycolysis during immune cell activation, suggesting important roles for not only hypoxia-inducible factor 1α (HIF1α) [41] and NFκB [45], but also TANK-binding kinase 1 (TBK1), inhibitor of NFκB kinase ε (IKKε) and hexokinase 2 [40].

Aside from its role in the adaption of activated immune cells to fit with particular energy demands of the tissues they move into, glycolysis seems to be involved in further cell line-specific signaling pathways promoting inflammation and immune cell differentiation [43]. For example, it was shown in macrophages that the pyruvate kinase isoenzyme M2 (PKM2), which normally catalyzes the last step of glycolysis and thus regulates its flux, is able to translocate into the nucleus, where it interacts with HIF1α. This results in an upregulation of HIF1α-dependent genes, including IL-1β [41,43,51,52]. Inhibiting this PKM2 translocation into the nucleus results in a switch of the macrophage from the pro-inflammatory M1 phenotype to the rather anti-inflammatory M2 phenotype. In addition, it was shown that the production of IL-1β in macrophages is increased by another mechanism closely connected to glycolysis. Moon et al. demonstrated that the glycolytic enzyme hexokinase is an activator of the nucleotide-binding domain (NOD)-, leucine-rich repeat (LRR)- and pyrin domain-containing 3 (NLRP3) inflammasome, which in turn regulates caspase-1 [53]. Caspase-1 than produces IL-1β, IL-18 and may induce pyroptosis.

Consistent with these findings, another crosslink between glucose metabolism, inflammatory status and phenotype of immune cells was demonstrated in T cells [54,55,56]. In addition, it was shown that the inhibition of glycolysis is able to induce the conversion of T helper 17 (T_H_17) cells to regulatory T (T_reg_) cells [57]. Thus, a HIF1α–dependent transcriptional program seemed to determine the glycolytic activity [57]. HIF1α in turn required signaling through mechanistic target of rapamycin (mTOR), making mTOR a key regulator of cellular metabolism [57]. At the same time, mTOR was found to compromise both survival and lineage stability in T_reg_ cells [40,43,58]. Another seminal finding concerning metabolic immune regulation is that the central glycolytic enzyme glyceraldehyde 3-phosphate dehydrogenase (GAPDH) seems to control the translation of interferon-γ (INFγ) in T helper 1 (T_H_1) cells [59,60]. While GAPDH binds and thus blocks INFγ-encoding mRNA in resting T_H_1 cells, this blocking effect is weakened in activated T_H_1 cells. This is due to the fact that an increased glycolytic flux causes a dissociation of GAPDH from the mRNA, resulting in an enhanced translation of the INFγ-encoding mRNA[61].

Combining all of this, hyperglycemia seems to be an adequate stress reaction on one hand, ensuring not only the energy supply of critical organs in systemic inflammation but also enabling the immune system to enter in a pro-inflammatory state and to carry out its designated functions. On the other hand, once a systemic inflammation becomes dysregulated, it might fuel the vicious circle leading to multi-organ dysfunction and death. Thereby, reactive intermediates such as ROS, RNS, and RCS might play a pivotal role.

### 2.4. Formation of ROS and RNS in Sepsis

As part of the innate immune response to pathogens, phagocytes such as neutrophils or macrophages start to produce massive amounts of superoxide anions (O_2_^−^) and hydrogen peroxide (H_2_O_2_) in order to kill the invaders. Nicotinamide adenine dinucleotide phosphate (NADPH)-oxidases are a major source of these ROS during this process called “respiratory burst” [62]. This respiratory burst results in excessively increased oxygen consumption [63]. Moreover, ROS produced during the respiratory burst seem to act as paracrine agents, both activating adjacent endothelial cells but also altering their function [64,65]. In addition to a large variety of changes, endothelial cells in turn increase intracellular ROS and RNS production. In addition to an NADPH-oxidase-dependent production of ROS, the mitochondrial formation of ROS via the uncoupling of respiratory chain complexes seems to be of great importance [64,65]. Aside from an increased production of nitric oxide (NO) through the inducible NO-synthase (iNOS) [66,67], several other pathways perpetuate ROS production and inflammation. On the one hand, NO stimulates the mitochondrial formation of H_2_O_2_ and O_2_^−^ via the inhibition of the cytochrome-c oxidases [68]. On the other hand, H_2_O_2_ induces the upregulation of iNOS via an activation of NFκB [69]. Furthermore, NO reacts with H_2_O_2_ to create peroxynitrite and other RNS [70]. Moreover, there is evidence that the mitochondrial complexes I and IV are susceptible to a deterioration by RNS [71,72], which in turn further compromises mitochondrial respiration [73]. Consistent with these findings, different animal models support the finding of altered mitochondrial function in sepsis. Although an impairment of mitochondrial function can be observed in the heart [74,75], liver [76,77] and intestine [78,79], the pathophysiological relevance remains uncertain [32]. Furthermore, Brealy et al. found a reduced mitochondrial complex I activity in the skeletal muscle from biopsies of non-surviving septic patients in comparison to survivors and post-operative controls (at 24 h after ICU admission) [80]. Sjövall et al. observed increased mitochondrial respiration in platelets of septic patients [81,82]. However, oxidative phosphorylation became less effective in these platelets with regard to ATP production, which is due to uncoupling [81]. Correspondingly, the increase in mitochondrial respiration during the early phase of systemic inflammation was inversely correlated with survival [82]. It is important to note that ROS are not only essential microbicides [83] but also represent potent activators of the innate immune system [72,84,85,86,87,88,89,90]. Taking this into consideration, mitochondrial dysfunction in sepsis might be part of the stress response in systemic inflammation. From this point of view, the described shift from the mitochondrial citrate cycle to the cytosolic glycolysis as the predominant source of ATP might be both a sequela of impaired mitochondrial function as well as a necessary measure to permit mitochondrial uncoupling for fueling inflammation in order to fight against the pathogen. Thus, hyperglycemia-induced changes in mitochondrial function in addition to the production of ROS and RNS are facets of a reasonable stress response becoming dysregulated in sepsis. Consistent with this, it was shown that moderate levels of ROS act as second messengers in signaling cascades and gene regulation, including nuclear factor 2 (Nrf2), nuclear factor kappa B (NFκB) and activator protein 1 (AP-1). In comparison, high levels of ROS induce apoptosis or even cause necrosis [91,92,93,94,95,96,97]. 

### 2.5. Reactive Carbonyl Species (RCS)—An Overlooked Group of Reactive Metabolites

ROS and RNS are very unstable and their deleterious effects are limited to the location where they are created in addition to the direct surroundings. A third group of more stable but still highly reactive metabolites might help to complete the picture of systemic inflammation. Reactive carbonyl species (RCS) [16] are a heterogeneous group of low molecular carbonyls, which are able to interact with a variety of biomolecules, such as proteins, deoxyribonucleic acid (DNA) of phospholipids, to cause an increased formation of advanced glycation end-products (AGEs). These modification processes involve structural changes, which result in functional changes, dysfunction or a total loss of function of the original protein [98,99]. These damaging effects are comparable to those of ROS, whereas RCS are more stable and thus are able to act systemically [100]. Methylglyoxal (MG) represents a RCS with particular pathophysiological relevance. In mammalian metabolism, MG is formed predominantly as a side product of glycolysis by non-enzymatic degradation of triosephosphates, glyceraldehyde-3-phosphate (GAP) and dihydroxyacetonephosphate (DHAP) [100,101]. Usually, 0.05% to 0.1% of the glycolytic flux is converted into MG [102], resulting in concentrations of 50–150 nM in human plasma [103,104]. Since MG is produced proportional to the glycolytic flux, it is not surprising that elevated MG plasma levels can be found in diseases characterized by altered glycolytic flux (e.g., diabetes mellitus). Moreover, MG seems to be more than a simple surrogate for the unbalanced glucose metabolism in diabetes mellitus, since it is clearly associated with the appearance of diabetic sequelae such as nephropathy and retinopathy [105,106]. Additionally, Bierhaus et al. proved its causal contribution to the development of diabetic neuropathy [107]. 

### 2.6. MG-Derived Carbonyl Stress in Sepsis—Diagnostic Value, Prognostic Value and Main Source of Formation

With regards to MG-derived carbonyl stress in critical illness, we were able to show in an earlier study that septic patients feature significantly higher plasma levels of MG compared to postoperative controls and healthy volunteers [108]. Moreover, MG outmatched the established markers of inflammation and infection, such as procalcitonin (PCT), C-reactive protein (CRP), soluble cluster of differentiation (CD) 14 subtype and IL-6, with regards to early and effective detection of sepsis in that study. Furthermore, we identified MG as an independent predictor of mortality in sepsis [108]. Elevated MG plasma levels seem to be inevitable in the chain of reasoning, considering that hyperglycemia is a characteristic feature of sepsis and MG derives from metabolic disease states, which are characterized by an increased glycolytic flux. Consistent with this, some authors interpret human MG-production as an essential defense mechanism of host neutrophils against invading bacteria [109]. However, other studies have shown that bacteria (e.g., *Escherichia coli*) also produce MG [110,111,112,113] and might therefore be the main source of carbonyl stress in bacterial sepsis. MG production via a specialized MG-synthase enables some *E. coli* strains to temporarily control their carbon flux [113]. Through this method, these bacteria are able to limit the stress of phosphate intermediates in carbon-rich environments, which allows these cells to grow for a limited time. If the environmental conditions change again to being less carbon-rich, these *E. coli* strains are able to survive due to this method, otherwise these strains die due to the accumulation of MG. Thus, MG-production embodies a high-risk ecological niche for these bacteria as it provides time for adaptation in unfavorable surrounding conditions [113]. It is still unknown if the main part of the observed MG-kinetics in patients suffering from septic shock are of endogenous or exogenous origin. However, data from our own workgroup strongly support the hypothesis arguing in favor for an endogenous source of carbonyl stress during systemic inflammation. This is due to the fact that, in addition to elevated MG plasma levels in postoperative controls of the above mentioned study [108], patients following liver transplantation [114] and patients suffering from severe trauma [115] are also characterized by significantly increased MG plasma levels. Both these patient groups clearly feature settings of sterile inflammation. Future research projects need to determine whether MG has a causal impact on the course of disease or whether it is just an epiphenomenon following stress hyperglycemia. However, a causal contribution of MG to the detrimental effects of a dysregulated systemic inflammation seems to be reasonable.

### 2.7. Pathophysiology of MG-Derived Carbonyl Stress in Sepsis

Under physiological conditions, MG reacts with modified arginine, lysine and cysteine residues in proteins [116]. Of these proteins, arginine seems to be the most susceptible. Ahmed et al. demonstrated that MG modifies human serum albumin in vivo [98]. In 92% of the albumin modifications, arginine residues are modified resulting in the formation of hydroimidazolone *N*^δ^-(5-hydro-5-methyl-4-imidazolon-2-yl)-ornithine with a loss of the positive charge of the side chain. Furthermore, they were able to localize a hotspot of modification at Arg-410 via tryptic peptide mapping. Although the clinical relevance of these findings is not clear, it is noteworthy that Arg-410 is located at the drug-binding site II and thus represents the active site of albumin-associated esterase activity [98]. Modified albumin was shown to induce the synthesis and secretion of IL-1β in addition to macrophage colony stimulating factor in human monocytic THP-1 cells [117,118]. In parallel, MG-modified arginine residues act as chemoattractants triggering receptor-mediated endocytosis and degradation [119]. Additionally, Du et al. demonstrated that following MG-mediated oxidation, the protein serine/threonine kinase “Raf-1”, a crucial enzyme for cell growth and development, is recognized as misfolded, which in turn results in ubiquitination and proteasome-dependent proteolysis [120]. Further cell damage can result from the interaction of RCS with DNA, forming the nucleotide AGEs “GdG” and “MGdG” and resulting in strand breaks and point mutations [121]. Meanwhile, it was shown that the plasma concentration of MG and the blood levels of hydroimidazolone (MG-H1) are correlated with the degree of tissue damage and disease progression in diabetes mellitus [105,106,122,123,124]. It was hypothesized that this observed association between high MG/ MG-H1 levels and the progression of diabetic retinopathy and nephropathy [105,106] might be caused by microvascular damage. This theory is based on the formation of MG-H1 at critical functional sites in type IV collagen [125]. Dobler et al. showed that these modifications induce anoikis of endothelial cell and impair angiogenesis [99]. Similar mechanisms are described in mesangial cells [126] and peripheral neurons [127]. Moreover, concerning diabetic neuropathy, Bierhaus et al. [107] showed that the administration of a specifically designed MG scavenger (GERP_10_, [128]) lowers the plasma levels of MG and MG-H1 and is thereby able to reduce the severity of clinically apparent hyperalgesia.

Regarding critical disease states, the influence of carbonyl stress on mitochondria and its relevance for ROS formation might be of special interest. The most probable theory seems to be that ROS, RNS, and RCS are a self-perpetuating system in which one factor stimulates the other [100,129,130]. Aborno et al. found that MG concentrations were significantly augmented during hydrogen peroxide-induced necrotic cell death [131]. The underlying mechanism involves the inhibition of downstream glycolytic enzymes resulting in an accumulation of glyceraldehyde 3-phosphate (GA3P) and dihydroxyacetone phosphate (DHAP) [132]. Conversely, decreased MG concentrations are associated with decreased ROS levels [131,133]. At the same time, RCS might profoundly impair mitochondrial function. For example, Morcos et al. suggested that mitochondrial proteins feature a particular vulnerability to MG-derived carbonyl stress [128]. In addition, Chan et al. showed that MG treatment increases ROS levels in osteoblasts and induces apoptosis via the mitochondrial pathway [134]. Likewise, Seo et al. treated human hepatic cells in vitro with MG and demonstrated a significantly increased permeability of the mitochondrial membrane under treatment [135]. Consistent with this, they found increased markers for liver cell damage (e.g., alanine transferase (ALAT) and aspartate aminotransferase (ASAT) in mice following treatment with MG. However, the transferability of their results into a clinical setting needs to be analyzed due to high treatment doses in vitro (3 and 10 mM) and in vivo (400 mg/kg bodyweight) [135].

In summary, MG-derived carbonyl stress might significantly alter crucial factors of the immune response to infection. Thus, it might influence not only the effector functions of immune cells but also the integrity of the endothelium and basic metabolic pathways, such as glycolysis and mitochondrial respiration. As a result, it might accelerate the development of sepsis-induced multi-organ dysfunction. However, the quantitative impact and the clinical relevance for the progression of the disease remain unclear and need to be specified.

### 2.8. Regulation of MG-Derived Carbonyl Stress in Sepsis

**Figure 2:**
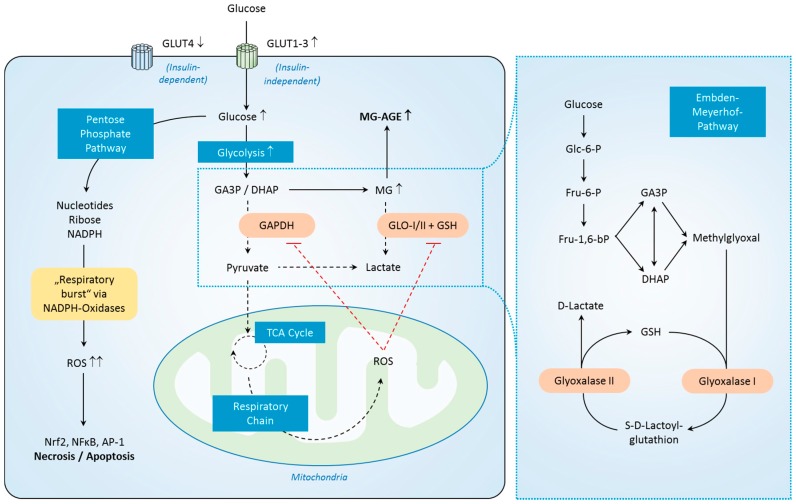
Regulation of MG-derived carbonyl stress in sepsis. Aside from enhanced glycolysis, increased formation of ROS with subsequent inhibition of GAPDH and the glyoxalase system in addition to glutathione consumption contributes to MG-derived carbonyl stress in sepsis. Small upward arrows symbolize increased levels. While solid arrows indicate pathway sequences, dashed arrows indicate reduced steps of a pathway. Abbreviations: GLUT = glucose transporter, MG = Methylglyoxal, MG-AGE = MG derived advanced glycation educts, GA3P = Glyceraldehyde 3-phosphate, DHAP = Dihydroxyacetone phosphate, GAPDH = Glyceraldehyde 3-phosphate dehydrogenase, GLO = Glyoxalase, GSH = Glutathion, TCA = tricarboxylic acid, ROS = reactive oxygen species, NADPH = Nicotinamide adenine dinucleotide phosphate, Nrf2 = Nuclear factor 2, NFκB = Nuclear factor κ B, AP-1 = Activator protein 1, Glo-6-P = Glucose-6-Phosphate, Fru-6-P = Fructose-6-Phosphate, Fru-1,6-bP = Fructose-1,6-Bisphosphate.

Mammals primarily detoxify MG via the glyoxalase pathway, which involves enzymes such as glyoxalase-I (GLO-I) and glyoxalase-II (GLO-II) [136,137]. These enzymes metabolize MG into lactate by using NADPH and glutathione [137,138]. Glyoxalase was first described in 1913 [139,140], but despite a hundred years of research [141], little is known about the actual mechanisms of its regulation. However, this way of MG detoxification seems to be severely disrupted in critical illness (Figure 2).

First of all, systemic inflammation is characterized by massive amounts of ROS, which need to be detoxified. However, a wide range of antioxidant mechanisms rely on glutathione as a co-substrate [142]. The resulting increased glutathione consumption consecutively impairs the glyoxalase system. Secondly, the ROS “hyperoxide” is known to directly inhibit GLO-1 [143]. Consistent with this, we previously found significantly increased levels of both ROS and RCS in critically ill patients compared to post-operative controls and healthy volunteers [108]. Moreover, in patients suffering from septic shock, the expression of GLO-1 was shown to be impaired [108]. These findings could be seminal, since the extent of GLO-1 expression was associated with the outcome of diabetes mellitus (DM) in rodent models [144]. It was postulated that the downregulation of GLO-1 is induced by an activation of the receptor for advanced glycation end-products (RAGE) [145,146,147,148]. Although the underlying pathway remains to be identified, it is well known that RAGE activates NFκB [149]. Moreover, GLO-1 expression has less downregulation in RAGE^(−/−)^ mice with DM than in controls [150]. Moreover, RAGE^(−/−)^ mice are characterized by having significantly improved survival in comparison to wild type controls in a murine model of abdominal sepsis (cecal ligation and puncture) [151]. Conversely, we found an elevated monocytic RAGE expression as well as significantly increased plasma levels of soluble RAGE (sRAGE) in both patients with septic shock [152] and patients following severe trauma [115]. Consistent with an increased MG-derived AGE formation during critical illness, these findings suggest a regulatory circuit involving MG-AGE-RAGE-GLO-1-MG, which results in deleterious formation of ROS, RNS, and RCS in systemic inflammation.

Recently, this hypothesis was challenged in the following way: Van Herreweghe et al. suggested that the regulation of GLO-I activity may be dependent on post-translational modification [153]. They demonstrated that protein kinase A (PKA) phosphorylates the active center of GLO-I and thus alters its function in fibrosarcoma cells. Following this phosphorylation, an accelerated formation of MG-derived AGEs as well as an increased rate of caspase-independent necrotic cell death was observed [153]. However, the informative value of these results is limited, since it is unknown to what extent the results in fibrosarcoma cells are representative for non-cancer cells in inflammation. To begin with, the activity of PKA is regulated by intracellular concentrations of cyclic adenosine monophosphate (cAMP). Cyclic AMP in turn represents the second messengers of many G-protein mediated hormone and cytokine responses [154] (e.g., induction of hepatic glycogenolysis after β-adrenergic stimulation [155]), processes of cell differentiation, cytoskeletal remodeling and proliferation in addition to apoptosis and immune modulation [156]. While AGE formation is thought to be part of pro-inflammatory signaling cascades (via RAGE and NFκB), recent in vivo studies suggest that globally high cAMP concentrations have moderate immunosuppressive effects [157,158]. Furthermore, cAMP-impaired neutrophil chemotaxis in vitro [159] decreased the release of histamine and leukotrienes from basophils or mast cells [160], which significantly reduced ROS formation [161,162]. Since intracellular cAMP measurements in septic patients are hard to perform, little is known about global cAMP levels in sepsis. Increased levels can be assumed in cells harboring adrenergic receptors, whereas cAMP-associated immunosuppressive effects obviously do not prevail, especially in the early phase of sepsis. Thus, it seems questionable whether cAMP levels are globally elevated in sepsis and the observed activation of PKA in fibrosarcoma cells (which in turn downregulates GLO-1 by phosphorylation) [153] can be extrapolated entirely to all cell types of septic patients. It instead appears that cAMP/PKA-dependent GLO regulation in sepsis needs to be evaluated in a more sophisticated manner, since cAMP/PKA signaling is known to be very specific for different tissues, cells and even subcellular units [163,164]. Specific A kinase anchoring proteins (AKAPs) direct PKA signaling to the desired site of action [165]. For NFκB, both stimulating and inhibitory mechanisms of PKA action have been identified [166]. In conclusion, both a RAGE-dependent as well as a PKA-dependent GLO-I regulation might be of value in sepsis. However, a detailed investigation needs to further illuminate the possible interactions and clinical relevance of these pathways. It is important to note that Morgenstern and colleagues recently confirmed the existence of an alternative pathway for the detoxification of MG in GLO-deficient neuronal Schwann cells [167]. In this study, they demonstrated that a knockout of GLO-1 is associated with a compensatory increase of MG detoxification via aldo-keto reductases (AKR), specifically AKR1b3. Whether this mechanism plays a relevant role in systemic inflammation remains to be clarified in further studies.

## 3. Conclusive Remarks

In summary, sepsis is not only characterized by mass production of cytokines and a dysregulated immune reaction, but also features a significant metabolic stress response. Amongst others, this includes drastic neurohumoral changes, profound alterations in glucose utilization, and a massive release of ROS, RNS, and RCS. Mitochondria might be of major importance in that context, although it is not absolutely clear whether their altered function is the result or the origin of reactive metabolites, such as ROS, RNS, and RCS. In a similar manner, sepsis-associated hyperglycemic metabolic states impair mitochondrial function via the formation of reactive metabolites but are necessary to maintain energy supply in times of impaired mitochondrial function. MG represents a highly reactive RCS and was shown to exhibit a distinct increase in the first hours of sepsis, making MG a more suitable discriminatory marker between patients with sepsis and patients suffering from sterile inflammation in comparison to conventional inflammation markers. Although MG was causally linked to late diabetic sequelae, there is no strong evidence that MG is linked to disease patterns of systemic inflammation and sepsis. However, since MG formation is closely linked to ROS and RNS formation, a causal contribution to the progression of the disease seems to be reasonable. Thus, a modulation of MG-derived carbonyl stress could be a suitable approach for the development of new therapeutic options in sepsis. This could be either realized via a direct scavenging of MG (e.g., GERP-10) or indirectly via a modulation of glyoxalase activity. However, the latter presumes a more detailed knowledge about the (patho)physiology of the glyoxalase-pathway in sepsis, which needs to be addressed in future research projects.

## Figures and Tables

**Table 1:** Overview of the most dominant hormonal changes in systemic inflammation and their contribution to the observed metabolic changes. Upward arrows symbolize “upregualation”, downward arrows symbolize “downregulation”.

Hormone	Pathway	Metabolic Changes
**Increased**		
**Cortisol**	− binding to the glucocorticoid receptor in the nucleus → expression of enzymes involved in gluconeogenesis ↑ and β2-adreno receptors ↑ and further anti-inflammatory proteins (e.g., lipocortin, Interleukin-1-Receptor Antagonist (IL-1RA), I κB Kinase) − inhibition of transcription of nuclear factor κ B (NFκB)-dependent genes	− gluconeogenesis ↑ in hepatocytes − lipolysis ↑ proteolysis ↑
**Nor-/Epinephrine**	− via β2-receptors (cAMP ↑) in liver and skeletal muscle − via β3-Receptors (cAMP ↑) in adipocytes	− lipolysis ↑ and gluconeogenesis ↑ in liver and skeletal muscle. − lipolysis ↑ and ketogenesis ↑ in adipocytes
**Vasopressin**	− via V1-receptors	− glycogenolysis ↑
**Insulin** (although its effects are impaired by peripheral insulin resistance)	− binding to the insulin receptor → activation and deactivation of enzymes via kinase cascades involving phosphoinositid-3-kinase, the PI3-cascade and the activation of protein kinase B (PKB).	In hepatocytes and skeletal muscle − insertion of glucose transporter type 4 (GLUT4) molecules into the cell membranes of adipocytes and skeletal muscles − glycogen synthesis ↑ − gluconeogenesis ↓ − triglyceride synthesis ↑ − lipolysis ↓ − cell growth ↑ − cell proliferation ↑ − autophagy ↓
**Glucagon**	− binding to G protein-coupled receptors− → cAMP ↑ → protein kinase A activity ↑	− glycogenolysis ↑
**Reduced**		
Thyroid-stimulating hormone (TSH) ↓, triiodothyronine (T3) ↓, thyroxine (T4) ↓	− changes of gene expression	− insertion of β-adreno receptors in cell membranes ↓ − insertion of α-adreno receptors in cell membranes ↑ − insulin secretion ↓ − corticosteroid and catecholamine production and secretion ↓
